# Extraction and Standardization of Patient Complaints from Electronic Medication Histories for Pharmacovigilance: Natural Language Processing Analysis in Japanese

**DOI:** 10.2196/11021

**Published:** 2018-09-27

**Authors:** Misa Usui, Eiji Aramaki, Tomohide Iwao, Shoko Wakamiya, Tohru Sakamoto, Mayumi Mochizuki

**Affiliations:** 1 Division of Hospital Pharmacy Science Graduate School of Pharmaceutical Sciences Keio University Tokyo Japan; 2 Social Computing Lab Graduate School of Information Science Nara Institute of Science and Technology Nara Japan; 3 Holon Co, Ltd. Hiroshima Japan; 4 Division of Hospital Pharmacy Science Faculty of Pharmacy Keio University Tokyo Japan; 5 Department of Pharmacy Keio University Hospital Tokyo Japan

**Keywords:** adverse drug events, natural language processing, medical informatics, medication history, pharmacovigilance

## Abstract

**Background:**

Despite the growing number of studies using natural language processing for pharmacovigilance, there are few reports on manipulating free text patient information in Japanese.

**Objective:**

This study aimed to establish a method of extracting and standardizing patient complaints from electronic medication histories accumulated in a Japanese community pharmacy for the detection of possible adverse drug event (ADE) signals.

**Methods:**

Subjective information included in electronic medication history data provided by a Japanese pharmacy operating in Hiroshima, Japan from September 1, 2015 to August 31, 2016, was used as patients’ complaints. We formulated search rules based on morphological analysis and daily (nonmedical) speech and developed a system that automatically executes the search rules and annotates free text data with *International Classification of Diseases, Tenth Revision* (ICD-10) codes. The performance of the system was evaluated through comparisons with data manually annotated by health care workers for a data set of 5000 complaints.

**Results:**

Of 5000 complaints, the system annotated 2236 complaints with ICD-10 codes, whereas health care workers annotated 2348 statements. There was a match in the annotation of 1480 complaints between the system and manual work. System performance was .66 regarding precision, .63 in recall, and .65 for the F-measure.

**Conclusions:**

Our results suggest that the system may be helpful in extracting and standardizing patients’ speech related to symptoms from massive amounts of free text data, replacing manual work. After improving the extraction accuracy, we expect to utilize this system to detect signals of possible ADEs from patients’ complaints in the future.

## Introduction

### Background

Adverse drug events (ADEs) are any untoward injuries resulting from the use of a drug [1]. They occur in around 18% of inpatients [1-4] and are a significant burden on health care and society. The ADEs are a cause of morbidity, and mortality and their economic loss is estimated at US $177.4 billion annually in the US [5]. In the field of pharmacovigilance, postmarketing surveillance such as spontaneous reporting is important for the detection of ADEs because clinical trials have limitations including patient sample size, population, and administration period [6].

The need to understand patients’ subjective complaints and to use other sources in pharmacovigilance has increased. Unlike health care providers, patients use various expressions and terminology to describe their situations. Direct reporting from patients is helpful in understanding their detailed symptoms and impacts on quality of life, which medical professionals tend to overlook [7-9]. For example, analysis of the content of comments posted on patients’ online community pages revealed unknown long-term symptoms of antidepressant withdrawal [10]. The Maintenance and Support Services Organization developed the Patient-Friendly Term List [11] based on the most frequent ADEs reported by patients and consumers to facilitate direct patient reporting of ADEs to regulators and the pharmaceutical industry. Despite its importance, little work has been done on exploring patient records until recently due to their unstructured, time-consuming data format.

Natural language processing (NLP) is the automatic manipulation of natural language such as narrative text and speech for extraction and structuring [12]. Numerous attempts have been made to use NLP in electronic health records (EHRs), social media, medical literature, or existing reporting systems [13-29]. Those studies found that NLP could identify various points for the assessment of medications (eg, inactive medication, nonadherence, patients’ mentions of ADEs).

In Japan, text analysis and automated detection of medical events from EHRs have been reported [30], and a tool for disease entity encoding was developed [31]. However, these 2 studies intended only to manipulate clinical text provided by health care professionals using medical terminology. No previous study dealt with patients’ complaints in their own words in Japanese.

### Prior Work

Nikfarjam et al [25] introduced a machine learning-based extraction system using conditional random fields (CRFs) for user posts on DailyStrength (precision: .86, recall: .78, F-measure: .82) and Twitter (precision: .76, recall: .68, F-measure: .72) to detect adverse drug reaction (ADR) signals. Freifeld et al [28] classified Twitter posts (precision: .72, recall: .72) to compare product-event pairs with the US Food and Drug Administration Adverse Event Reporting System (FAERS) data.

In the mining of patients’ reports, Topaz et al [26] used a linguistic-based approach comparing EHRs (clinicians’ reports) and social media (patients’ mentions) for 2 common drugs. White et al [27] used search log data for the identification of ADE signals and a comparison with FAERS data resulted in high concordance as determined by the Area Under the Curve Receiver Operating Characteristics curve of .82. Denecke et al [29] collected data from multiple media sites with keyword lists and classified texts as relevant/irrelevant using support vector machines.

Although no previous studies have been completed in Japanese, Aramaki et al [30] reported on a system to extract medical event information from Japanese EHRs based on CRFs (precision: .85, recall: .77, F-measure: .81). The text source in their study was written in medical terminology, mainly by physicians. No lexicon to standardize patients’ informal expressions such as the Patient-Free Term List [11] and the work of Freifeld et al [28] has been published in Japanese.

### Study Aim

This study aimed to develop techniques to establish a method for extracting and standardizing patient complaints from electronic medication history data (EMHD) accumulated in a Japanese community pharmacy for the detection of possible ADE signals.

## Methods

### Concept of the System

**Figure 1:**
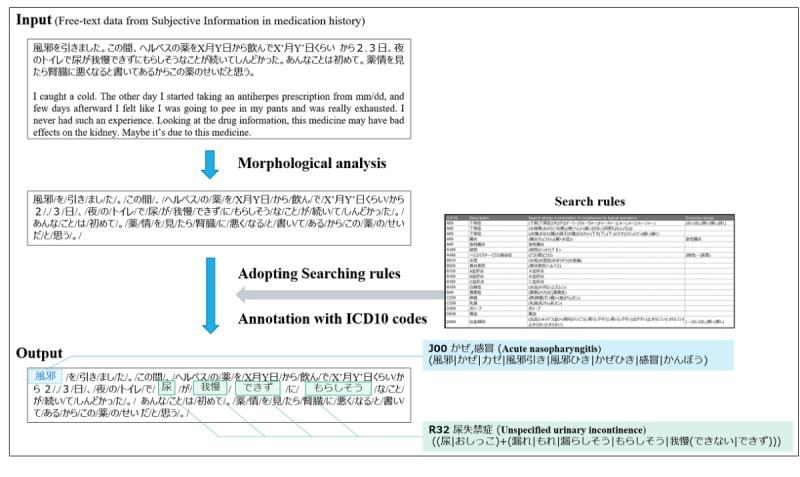
Concept of the system. ICD-10: *International Classification of Diseases, Tenth Revision.*.

We propose a system that automatically extracts and standardizes patient complaints (Figure 1). In this system, subjective information included in the medication histories collected from a pharmacy is input data, and data in which *International Classification of Diseases, Tenth Revision* (ICD-10) codes are attached to patient expressions are outputs. A dictionary-based method was adopted for extraction and standardization. The processing steps in the system are as follows. First, morphological analysis is performed on input data. Next, the search rules are applied to split data. In the search rules, morpheme combinations in general expressions and the corresponding ICD-10 codes are described for each line, and exclusion rules are set for some ICD-10 codes. When a patient expression satisfies the search rules, a corresponding ICD-10 code is given. Procedures for creating the search and exclusion rules and system development procedures are detailed in “Search Rules” and “System Development.”

### Data Sources

The EMHD stored in a community pharmacy were used as the source of patients’ comments. When pharmacists dispense prescription drugs to patients, they are required to record the results of medication instructions and patients’ queries/responses. A medication history in Japan is typically written in the “SOAP” format, which consists of 4 sections: “Subjective information” (complaints of the patient), “Objective information” (objective indicators such as laboratory findings or names of drugs prescribed), “Assessment” (the pharmacist’s findings on the occurrence of ADRs, interactions, or doubt about prescription instructions), and “Plan” (action plan of the pharmacist derived from the assessment).

Although patients do not write the medication history, of those 4 sections the “Subjective information” appeared to be the most appropriate text source, because pharmacists complete that section in the patients’ own words.

Patients’ comments were extracted from the EMHD of a community pharmacy operated by Holon Co, Ltd, Hiroshima, Japan. This company operates a chain of 14 pharmacies, and the data used in this study mainly came from a single one. The study period was from September 1, 2015 to August 31, 2016. Personal information such as patients’ names and birth dates were anonymized before analysis.

Information on the hospitals or clinics that issued prescriptions for which the subjective information used in this study was derived is shown in Table 1. The pharmacy filled a total of 42,120 prescriptions during the study period for the top 9 prescribing hospitals or clinics. The number of prescriptions from medical institution A was the highest (18,273/42,120, 43.5%). Clinic A specializes in otolaryngology, and the patients are older adults who often complain of dizziness or hearing loss.

**Figure 2:**
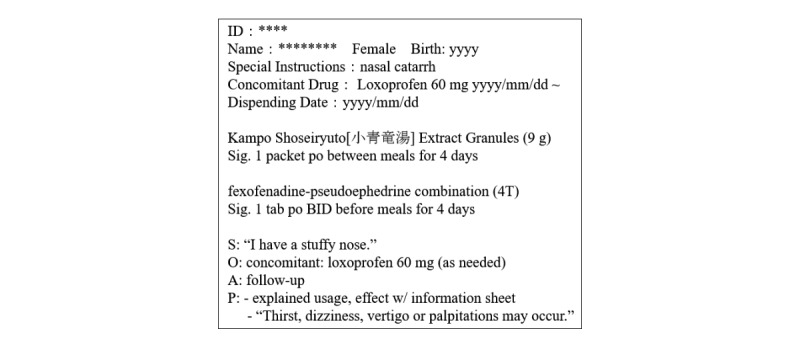
Example of a medication history.

Table 2 shows the items recorded in the EMHD, while Figure 2 is an example of a recording object.

**Table 1:** Backgrounds of the prescriptions used in this study (N=42,120).

Hospital/ clinic	Specialty	Main patient characteristics	Prescriptions, n (%)
A	Otolaryngology	Elderly Dizziness Tinnitus Hearing loss	18,273 (43.38)
B	General medicine; cardiology	Elderly Heart disease Hypertension	5356 (12.72)
C	General medicine; gastroenterology; cardiology	Elderly Digestive tract diseases Circulatory diseases	537 (1.27)
D	General medicine; Kampo^a^ medicine	Elderly Kampo^a^ medicines (for >half of the patients)	989 (2.35)
E	Neuropsychiatry	Wide range of age-groups	377 (0.90)
F	Neuropsychiatry	Wide range of age-groups	563 (1.34)
G	Obstetrics and gynecology	Gynecological conditions	649 (1.54)
H	Obstetrics and gynecology	Infertility treatment	4206 (9.99)
I	Breast surgery	Breast cancer patients visiting for diagnosis and postoperative care	2608 (6.19)
Other^b^	Various	Various	8562 (20.33)

^a^The word “Kampo” means herbal medicine in Japanese. The term “Kampo Shoseiryuto” is commonly used to treat watery nasal discharge, nasal congestion, watery sputum, and sneezing.

^b^These are medical institutions that are not the major clinics “A” to “I” from which this pharmacy receives prescriptions.

**Table 2:** Items recorded in the electronic medication history data.

Category			Content
Identification			Identification of patient
Name			Name of patient
Sex			Male/female
Birth^a^			Year/month/day
Special instructions			Notes as required (eg, disease entity)
**Concomitant drugs**			
	Drug name	YJ-code^b^ or product name	
	Dosing period	Year/month/start date/end date	
**Prescription data**			
	Dispensing date	Year/month/day	
	Drug name	YJ-code or product name	
	Dosage/administration^c^	Free text	
**Medication counseling**			
	Counseling date	Year/month/day	
	Subjective information^d^	Free text	
	Objective information^e^	Free text	
	Assessment^f^	Free text	
	Plan^g^	Free text	

^a^Only the year of birth data was used.

^b^YJ-codes identify prescription drugs covered by insurance in Japan.

^c^Not used in this study.

^d^Includes patient complaints.

^e^Includes laboratory data.

^f^Includes pharmacists’ assessments of patient conditions.

^g^Includes pharmacists’ plans for prescription questions, patient education, and follow-up.

### Search Rules

We created search rules to identify the appropriate ICD-10 code from the free text in the “Subjective information” section and developed a coding system that annotates the ICD-10 codes within patient complaints. The ICD-10 was originally an English-based system but is also used in Japan. It was translated into Japanese by the World Health Organization, and a coding rulebook was published. For example, in Medis [32] the ICD-10 is given as the basic classification code, and coding matched as closely as possible to clinical interpretation is undertaken. Although it may be possible to use the Medical Dictionary for Regulatory Activities (MedDRA) or the International Classification of Primary Care as a medical code system, we adopted ICD-10 in this study because it is used for insurance claims in Japan and because many coders are familiar with ICD-10.

In developing the system, a nurse with 10 years of experience in the field of terminal care and a medical coder with 20 years of experience created the search rules based on the expressions in the “Subjective information” section. A programmer read the search rules and developed a program to accommodate new expressions. Search rules were created by a combination of morphological analysis and common expressions.

The search rules govern the pattern for analyzing comments included in the subjective information. The rules were saved in Microsoft Excel format with the corresponding disease entity category and ICD-10 codes. For example, to search for “D69.9: Hemorrhagic condition, unspecified,” the search strings are “(出血|しゅっけつ|血)+(傾向|けいこう|し易く|しやすく|し易い|しやすい|出やすい|止まりにくい|とまりにくい|止まらない|とまらない).” In English, this would translate to “(bleeding|blood)+(tendency|easy to|hard to stop|won’t stop|not stop).” Written Japanese utilizes 3 orthographic systems: Chinese characters, *hiragana*, and *katakana*. Therefore, the actual search strings are longer than in English. All rules are shown in Multimedia Appendix 1. The rule-making steps are shown in Textbox 1. We repeated this process 5 times over 1 month in order to refine the search rules.

The nurse first checked the free text recorded in the “Subjective information” section and selected complaints referring to patients’ symptoms. Then words related to ICD-10 codes were manually extracted from the complaints. Finally, the extracted words were added sequentially to the search string for each ICD-10 code. The search strings consist of patterns of word combinations using “|” (logical sum) or “+” (logical product). At present, a maximum of 3 words/terms can be combined in a string separated by “+” signs. For example, from the text “blood pressure today was a little high,” the terms “blood pressure” and “a little high” were extracted, and the system annotated the text with the ICD-10 code “I10: hypertension.”

However, some text found in the “Subjective information” section could not be annotated with an ICD-10 code even though it followed the search rules. Therefore, we set exclusion rules for some codes, which were created following the same procedure as for the search rules but were only applied when a health care worker could visually confirm the keyword for exclusion. For the previous example of “D69.9: Hemorrhagic condition, unspecified,” terms with “(-|ない|なし|無い|無し),” in English, “(-|no|none|negative|never|don’t)” were excluded even if they included search strings. For example, “(血がとまりにくい),” in English, “(the bleeding won’t stop),” was annotated as D69.9, but “(血がとまりにくいことはない),” in English, “(I never felt the bleeding wouldn’t stop),” was excluded.

### System Development

The system developed extracts complaints related to patients’ symptoms from the “Subjective information” section of EMHD automatically and annotated each complaint with the ICD-10 code using the search rules above. During system development, we used Perl as the programming language and MeCab [33] as a morphological analyzer. The Microsoft Excel format was used for subsequent analysis.

The development procedure can be summarized as follows:

1. Subjective information was extracted from each saved Microsoft Excel file
2. Morphological analysis was performed to extract subjective information, separating the text with spaces into minimum meaningful units of words/terms
3. After the processes above were performed, the subjective information was copied back into a Microsoft Excel file. Search rules and exclusion rules were applied to the subjective information by analyzing each complaint and searching for the ICD-10 code
4. If an appropriately matching ICD-10 code was found, the complaint was annotated with the ICD-10 code and the corresponding disease entity

The coding system adapts the search rules (shown in Multimedia Appendix 1) in order from the top. If an adaptable rule is found, the result of ICD-10 coding is output. If multiple rules are matched, all of them are output in the results.

**Rule-making steps.**

1. Make seed rules
2. Apply seed rules to development set
3. Error analysis performed by two rule curators (the nurse and medical coder)
4. New rules added by the programmer, who converts the error analysis to general expressions
5. Repeat steps 3 and 4

**Figure 3:**
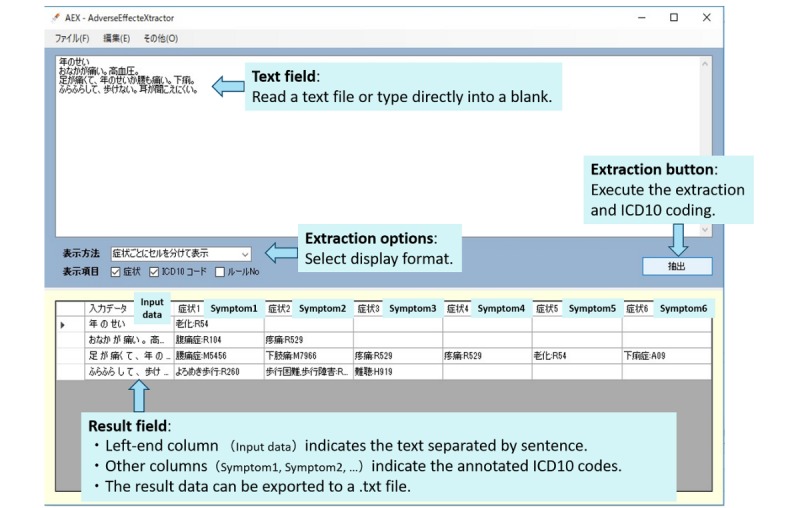
System interface screenshot. ICD-10: *International Classification of Diseases, Tenth Revision*.

### Optimization of System Performance

For optimal performance of the system, the system-annotated disease entities should ideally match the entities manually annotated by health care professionals. As mentioned above, the more thoroughly the search rules are satisfied, the more accurate the system. Therefore, we reviewed the search rules multiple times to determine the most appropriate ones to improve the accuracy of the system.

In this study, we did not attempt machine learning for the detection of relevant terms to match ICD-10 codes. By adding search rules as appropriate, free text can be automatically associated with ICD-10 codes via the system.

### Experiment

An evaluation experiment was conducted to confirm the performance of the system. Five thousand complaints from the subjective information were processed, and 323 search rules were created. In the experiment, health care workers (1 nurse and 1 pharmacist) first independently annotated the 5000 complaints manually with the ICD-10 codes. Second, 108 mismatched annotations were excluded, and the data from the remaining 2348 were used as correct answers for the subsequent step. Finally, the system with 323 search rules was applied to the 5000 complaints.

The subjective information used in this study consisted of multiple sentences, and thus several patient expressions were obtained from one “Subjective information” section. Since each patient expression is linked to the ICD-10 code, multiple ICD-10 codes are assigned to a single “Subjective information” section. In evaluating the system in this study, if one of the plural ICD-10 codes differed from the manual result, it was judged that all other coding for that entry was incorrect (unmatched). Figure 3 shows an actual system execution screen.

Based on the results of this experiment, the precision, recall, and F-measure of the system were calculated [34,35]. Precision was calculated by dividing the matched number (the number of “Subjective information” sections for which manual coding and system coding had the same result) by the searched number (the number of “Subjective information” sections that the system annotated with ICD-10 codes). Recall was calculated by dividing the matched number by the correct answers (the number of “Subjective information” sections manually coded). The F-measure was calculated by taking the harmonic mean between precision and recall.

### Ethical Considerations

This study was approved by the Ethics Committees on Human Research of the Faculty of Pharmacy, Keio University and Nara Institute of Science and Technology.

## Results

Examples of correct answer data and system execution results are shown in Table 3. From 5000 complaints, 2348 ICD-10 codes were extracted by health care workers. The system extracted 2236 codes, 1480 of which matched the manual results. The system performed .662 for precision, .630 for recall and .646 for F-measure. Table 4 shows precision and recall for the 10 most frequent symptoms extracted by health care workers.

**Table 3:** Comparison between manual and system extraction of ICD-10 codes for patient complaints from typical examples.

Patient complaints (Original text of Japanese is attached)	Manual results	System results	Matching
めまい は 起こっ て い ない です 。 暑 さ で フラー っと する こと が ある 。 No vertigo/dizziness. Sometimes I feel unsteady due to the heat.	R42: Dizziness and giddiness; R26.0: Ataxic gait	R42: Dizziness and giddiness	Not matched
昨日 の 夜 から 頭 の 後ろ が 重い よう な 感じ が する 。 風邪 か と 思っ て 受診 。 From the night before last, the back of my head felt heavy. I probably caught cold, so I saw a doctor.	J00: Acute nasopharyngitis (common cold); R51: Headache	J00: Acute nasopharyngitis (common cold); R51: Headache	Matched
私 よく 走っ ちゃう ん だ けど 坂 で 躓い て ね 。 ちょっと な のに 大きな アザ ガ でき た 。 整形 外科 の 先生 は 血 流 の 薬 が ある から って 。 で も 飲ま ない と だめ だ と いわ れ た 。 I was in a hurry, stumbled, and fell. A big bruise came up. My orthopedist said it may be caused by my blood thinner, though I have to continue that medicine.	Q82.5: Congenital nonneoplastic nevus	Q82.5: Congenital nonneoplastic nevus	Matched
血圧 は ちょっと 高かっ た の 。 １ ４ ０ 後半 。 家 だっ たら １ ３ ０ くらい なん だ けど 。 整形 外科 で もらっ た パップ は かぶれ て しまっ た 。 My blood pressure was a bit high, in the upper 140s, although it’s around 130 when I’m at home. I got a rash from plasters prescribed by orthopedics.	I10: Essential (primary) hypertension; L259: Unspecified contact dermatitis, unspecified cause	I10: Essential (primary) hypertension; L259: Unspecified contact dermatitis, unspecified cause	Matched
風邪 を 引き まし た 。 この間 、 ヘルペス の 薬 を X月Y日 から 飲ん で X’月 Y’日くらい から ２ ． ３ 日 、 夜 の トイレ で 尿 が 我慢 でき ず に もらし そう な こと が 続い て しんどかっ た 。 あんな こと は 初めて 。 薬 情 を 見 たら 腎臓 に 悪く なる と 書い て ある から この 薬 の せい だ と 思う 。 I caught a cold. The other day I started taking an antiherpes prescription from mm/dd, and few days afterward I felt like I was going to pee in my pants and was really exhausted. I never had such an experience. Looking at the drug information, this medicine may have bad effects on the kidney. Maybe it’s due to this medicine.	J00: Acute nasopharyngitis; R32: Unspecified urinary incontinence	J00: Acute nasopharyngitis; B02.9: Zoster without complication; R32: Unspecified urinary incontinence; R53: Malaise and fatigue; N28.9: Disorder of kidney and ureter, unspecified; R94.4: Abnormal results of kidney function studies	Not matched

**Table 4:** Precision and recall for the 10 most frequent symptoms.

Rank	ICD-10^a^	Matched, n (%)	Correct answer, n (%)	Searched, n (%)	Precision	Recall
1	R42 (Dizziness and giddiness)	146 (9.86)	173 (7.37)	254 (11.36)	.575	.844
2	J00 (Acute nasopharyngitis^b^)	189 (12.77)	208 (8.86)	226 (10.11)	.836	.909
3	R52.9 (Pain, unspecified)	108 (7.29)	146 (6.22)	199 (8.90)	.543	.740
4	F19.6 (Mental and behavioral disorders^c^)	142 (9.59)	168 (7.16)	197 (8.81)	.721	.845
5	R05 (Cough)	114 (7.70)	134 (5.71)	143 (6.40)	.797	.851
6	H931 (Tinnitus)	99 (6.68)	131 (5.58)	134 (6.00)	.739	.756
7	R26.0 (Ataxic gait)	41 (2.77)	59 (2.51)	120 (5.37)	.341	.695
8	I10 (Essential [primary] hypertension)	51 (3.44)	89 (3.79)	87 (3.89)	.586	.573
9	F51.1 (Nonorganic hypersomnia)	39 (2.63)	46 (1.96)	85 (3.80)	.459	.848
10	R53 (Malaise and fatigue)	40 (2.70)	59 (2.51)	81 (3.62)	.494	.678

^a^ICD-10: *International Classification of Diseases, Tenth Revision*.

^b^Common cold.

^c^Due to multiple drug use and use of other psychoactive substances.

**Six reasons for unmatched results. ICD-10: International Classification of Diseases, Tenth Revision.**

**1. Misdetection of negation or possible event**

System misread an expression including negation or possible event as a symptom that actually occurred (eg, “dizziness has not occurred,” “If I feel dizzy”)

**2. Misdetection of a clinical test item**

System mistook a clinical test term as a patient symptom (eg, “test for dizziness”)

**3. Misdetection of drug class name**

System mistook the name of the drug class as a patient symptom (eg, “painkiller” mistaken for “R529: Pain, unspecified”)

**4. Misdetection of unrelated words**

System mistook unrelated words as a patient symptom (eg, “I’m getting old” mistaken for “R54: Senility”)

**5. False negative**

System missed a word that indicates a patient symptom

**6. Inappropriate ICD-10 code**

System failed to choose the appropriate ICD-10 code even if it extracted words related to a patient symptom

The results indicated that the average performance of the system was .66 for precision, .63 for recall, and .65 for the F-measure. Comparing the performance for each symptom, the precision of “dizziness and giddiness,” “pain, unspecified,” and “ataxic gait” was especially low. We identified 6 reasons for the unmatched results for these 3 symptoms, as shown in Textbox 2.

Table 5 details the unmatched results and typical examples for 3 symptoms. The main reason for discordance between manual and system coding was misdetection of negation or possible event in “R42: dizziness and giddiness” (79/108 results, 73.1%) and “R26.0: ataxic gait” (71/79 results, 90%), whereas misdetection of drug class name was the most common in “R52.9: pain, unspecified” (28/91 results, 31%).

**Table 5:** Details of unmatched results and typical examples for 3 symptoms.

Symptom and category		n (%)	Example of Patient complaints (Original text of Japanese is attached)	Manual results	System results
**R42: Dizziness and giddiness (N=108)**					
	1 (Misdetection of negation or possible event)	79 (73.1)	眩暈 は 起き て い ない が 、 ２ 回 くらい ふらつき が あっ た 。 今日 Ｄｒ に 診 て もらっ たら 大丈夫 だ と 言わ れ た 。 I didn‘t feel dizzy but staggered about twice. Today I saw a doctor and be said there was no problem.	R26.0: Ataxic gait	R42: Dizziness and giddiness; R26.0: Ataxic gait
	2 (Misdetection of clinical test item)	5 (4.6)	なぜ か ニセルゴリン だけ １ ０ 錠 余っ てる 。 調子 は なんとも ない 。 眩暈 も 検査 し た けど 問題 ない と 言わ れ た し 、 聞こえ も 悪く なっ て ない と 言わ れ た 。 I don’t know why, but I have 10 leftover Nicergoline pills. I feel fine. I was examined for dizziness and no problem was found. I was also told that there was no problem with my hearing.	—	R42: Dizziness and giddiness; H91.9: Unspecified hearing loss
	3 (Misdetection of drug class name)	8 (7.4)	めまい の 薬 は 昼 飲め ない こと が 多く て 残り が ある の 。 ずっと 仕事 だ から 飲む の が 難しく て 。 I have leftover motion sickness medicine because I often don’t take it in the daytime. I work all day and it’s hard to take it.	—	R42: Dizziness and giddiness; R13:Dysphagia
	4 (Misdetection of unrelated words)	4 (3.7)	母 の 介護 で 忙しかっ た けど 秋 に 亡くなっ て 、 葬式 とか で また 忙しく て あまり 薬 が 飲め て なかっ た ので 残り は あり ます 。 めまい も そんなに ひどく なっ て なかっ た です 。 I was busy taking care of my mother. She passed away this autumn and I got busy with the funeral arrangements, etc. I didn’t have time, so there are still some pills. The dizziness didn’t get much worse.	R42: Dizziness and giddiness	R42: Dizziness and giddiness; R99: Other ill-defined and unspecified causes of mortality
	5 (False negative)	11 (10.2)	めまい は 起こっ て い ない です 。 暑 さ で フラー っと する こと が ある 。 No vertigo/dizziness. Sometimes I feel unsteady due to the heat.	R42: Dizziness and giddiness; R26.0: Ataxic gait	R42: Dizziness and giddiness
	6 (Inappropriate ICD10 code)	1 (0.9)	ふらふら する の は 最近 落ち着い てる 。 Ｄｒ に 言わ れ て 、 起き上がる とき も ゆっくり 起きる よう に し たら 眩暈 あまり 起き なく なっ た 。 The dizziness is getting better. My doctor told me to stand up slowly and when I tried that I didn’t feel dizzy.	R42: Dizziness and giddiness; H81.1: Benign paroxysmal vertigo	R42: Dizziness and giddiness; R26.0: Ataxic gait
**R52.9: Pain, unspecified (N=91)**					
	1 (Misdetection of negation or possible event)	20 (22.0)	血圧 １ １ ０ くらい 。 ふらつく こと は ない です 。 筋肉 痛 も ない です 。 気 に なっ た こと は ない が 、 言わ れ て みれ ば 、 たまに だるく なる こと が あり ます 。 BP is around 110. No dizziness and no muscle pain. But now that you ask, I sometimes feel malaise.	R53: Malaise and fatigue	M79.1: Myalgia; R52.9: Pain, unspecified; R53: Malaise and fatigue
	3 (Misdetection of drug class name)	28 (30.8)	痛く ない から 神経 痛 の 薬 は もう いら ない 。 納豆 が 本当は 大好き な ん だ けど 、 ワーファリン 錠 飲ん でる から 止め てる 。 納豆 関係 ない 薬 も ある らしい けど 、 値段 が 高い らしい ね 。 II don’t feel pain, so I don’t need the nerve medicine any longer. I really love natto (fermented soybeans), but have to avoid it because I take warfarin. I think there is some kind of medicine that is not affected by eating natto, but I hear it’s really expensive.	—	R52.9: Pain, unspecified; M79.2: Neuralgia and neuritis, unspecified
	4 (Misdetection of unrelated words)	8 (8.8)	耳鳴り と のど が 痛い 。 My ears are ringing and I have a sore throat.	H93.1: Tinnitus; J02.9: Acute pharyngitis, unspecified	H92.0: Otalgia; H92.1: Otorrhea; R52.9: Pain, unspecified
	5 (False negative)	9 (9.9)	胃 が 痛い 、 のん で も 治ら ん から 先生 に 相談 し た 。 腰 も い たい ん よ ね 。 ロキソニン は 手持ち 無くなっ た 。 今回 で て ない ？ あら 。 ボラザ Ｇ 軟膏 は ２ １ 本 持っ てる けど ちょっと 足り ない かも ね 。 My stomach hurts. I took some medicine but didn’t feel better so I saw a doctor. My lower back also hurts. I’m out of Loxoprofen, so did the doctor give me a new prescription? I have 21 tubes of ointment but that may not be enough.	R10.1: Pain localized to upper abdomen; R52.9: Pain, unspecified; M54.5: Low back pain	R101: Pain localized to upper abdomen; R52.9: Pain, unspecified
	6 (Inappropriate ICD10 code)	26 (28.6)	前 の 粉薬 を 止め たら 尿 の 出 は 治っ た ん だ けど 、 他 の 薬 を 続け て い たら 手の甲 と 関節 が ピリピリ 痛み 始め て 驚い た 。 飲む の を 辞め たら 治っ た よ 。 After I stopped taking the powdered medicine, my urine flow improved but I’m still taking the other medicine. I suddenly felt a sharp pain on the back of my hand and in the joints, but that stopped after I quit taking the medicine.	M25.5: Pain in joint; M79.6: Pain in limb	M25.5: Pain in joint; 52.9: Pain, unspecified
**R26.0: Ataxic gait (N=79)**					
	1 (Misdetection of negation or possible event)	71 (89.9)	カルデナリン 錠 が 中止 に なっ て 、 フラ ツキ は あれ っきり よく なっ た 。 血圧 の 薬 は 内科 で かるい の が 追加 に なり まし た 。 眼科 は 今 は かかっ て なく 、 内科 で 目薬 出し て もらっ て ます 。 After I stopped taking Doxazosin, the light-headedness got better. A new mild blood pressure medicine was prescribed by the doctor. I don’t go to the eye doctor anymore, so I have my regular doctor prescribe eyedrops.	—	R26.0: Ataxic gait
	4 (Misdetection of unrelated words)	2 (2.5)	かるい フラ ツキ ある ん だ けど 大丈夫 。 内科 は いっ て ない 。 先生 行け って 言わ ない から 大丈夫 な ん だ と 思う 。 セロクラール と パルトックス 錠 は わかる から 赤 線 は ひか なく て いい よ 。 I stagger a little bit, but that’s OK. I didn’t go to the Internal Medicine Department because my doctor didn’t tell me to, so it probably isn’t a problem. I know which pills are Cerocral and Pantethine, so you don’t have to draw a red line on the bottles for me.	R26.0: Ataxic gait	R26.0: Ataxic gait; R21: Rash and other nonspecific skin eruption
	5 (False negative)	5 (6.3)	血圧 は いつも より 良かっ た 。 病院 で 血糖 が ６ ８ だっ た から 飴 玉 食べ た 。 ブドウ糖 も 持っ てる 。 ふわっと する 症状 も あっ た 。 My blood pressure was better than usual. My blood glucose was 68, so I ate some candy. I carry glucose tablets with me, too. I felt weightless.	E16.2: Hypoglycaemia, unspecified; R26.0: Ataxic gait	R26.0: Ataxic gait
	6 (Inappropriate ICD10 code)	1 (1.3)	頭 が フラフラ する ので 医師 に 相談 し た 。 リリカ が 効き 過ぎ て いる の で は ない か と の こと 。 I felt woozy and went to the doctor. He thought that the Pregabalin was too strong.	R42: Dizziness and giddiness	R26.0: Ataxic gait

## Discussion

### Principal Results

Nikfarjam et al [25] and Aramaki et al [30] used CRFs, and Freifeld et al [28] used a tree-based dictionary matching algorithm for extracting the terms. Our approach involved rule-based searching, which is much simpler but less tolerant of orthographic variants. Additionally, differences in linguistic features might have contributed to the gap between the results of the present study and nonJapanese ones [25,28]. In written Japanese, words are not separated by spaces, and therefore the accuracy of extraction is affected by the quality of morphological analysis. Considering these points, the results are at least adequate as the first step in possible ADE signal detection.

This was the first attempt to standardize patients’ expressions with the Japanese version of ICD-10 and to use the “Subjective Information” section in the medication history as a source. The advantage of using the medication history is its structured format and data storability. The medication history is recorded for patient monitoring including side effects. Its features provide more specialized information relevant to possible ADEs than social media like Twitter or EHRs in hospitals. Moreover, the number of pharmacies in Japan is increasing [36,37], and pharmacists are required to record patient medication histories for health insurance claims. Thus, huge amounts of data on patients’ medication are available, making medication histories an appropriate source for ADE signal detection.

It is not necessary for ADEs to have causal relationships with drugs, whereas ADRs must have a reasonable association with drug use. Using patient records to detect ADRs is a major challenge because causality cannot be readily assessed; however, it is also important to detect potential ADE signals.

In this study, some text could not be annotated with ICD-10 codes. As compared with the performance of health care professionals, our newly developed system performed at levels of .66 for precision, .63 for recall, and .65 for the F-measure. These values are relatively lower than in previous studies [25,28,30], likely due to differences in methodology. As explained in the Experiment section, if one of the many ICD-10 codes was different from the manual result, all other coding was regarded as incorrect (unmatched) for that entry. This is one reason why the F-measure was lower than in previous research.

There was also insufficient specific information about the condition of each patient. Because the majority of patients are not medical experts, they describe their symptoms in everyday language, which is more equivocal and more inflected than medical terminology. Nikfarjam et al [25] reported similar aspects of ambiguity and lack of context in patients’ wording.

The dialect spoken can affect the subjective information, although, of 5,000 complaints analyzed in this study, only 7 were recorded in a regional dialect. This is probably related to the nature of the text. Although it is recommended that pharmacists record patients’ statements exactly, it is possible that they replace dialect expressions with standard wording to make the information easier to understand by others later.

Regarding standardization across languages, the present system could be applied to other languages to some extent by translating the morphemes used for the search rules or by adding or refining the search rules later.

### Limitations

There were some limitations of this study. First, qualitative differences in the text data could have occurred. The “Subjective information” section is filled in by pharmacists, and therefore they may interpret and summarize patients’ comments when they record them. To ensure that the medication histories of all patients are recorded during the daily business hours of community pharmacies, in some cases fixed-form complaint set phrases and excerpts of comments may be relied on to decrease the time needed to complete the “Subjective information” section. It is therefore possible that the finer nuances patients hope to convey are altered or lost during the process. Qualitative differences were also noted among pharmacists for the contents of the “Subjective information” section. Some wrote about symptoms using explicit medical terminology (eg, “back pain and knee pain were unabated”). Others included general information unrelated to symptoms (eg, greetings and general conversation transcribed word for word).

Second, it was difficult for the system to determine whether the extracted keyword was related to patients’ symptoms or those of others. For example, from the sentence “My friend had hypertension,” the system may extract “hypertension,” although it is unrelated to the speaker’s condition. This point should be improved by revising the search rules after consultation with regulatory experts or using machine learning to deal with ambiguity.

Also, since only 1 of 14 pharmacies in a single chain participated in this study, there is a possibility that the search rules were optimized for patients receiving prescriptions from specific medical departments. In the experimental results, the most frequent ICD-10 code was “dizziness and giddiness.” As shown in Table 1, the target pharmacy frequently dispenses prescriptions from otolaryngologists, and the results may reflect this potential bias. Before the practical application of the system, it is necessary to improve the search rules by considering a wider range of medication histories including data from other community pharmacies.

ICD-10 codes were used as normalization terms for patients’ complaints regarding their symptoms because they are widely available and understood, but MedDRA is thought to be more suitable for extracting information on ADRs and for signal detection. We are currently enhancing the system to accommodate MedDRA terms.

### Conclusions

In this study, we developed an automated system to extract terms related to symptoms from the verbal complaints of Japanese patients. As a result of an evaluation experiment comparing automated with manual extraction, the system performed at the level of .66 in precision, .63 in recall, and .65 for the F-measure. Although the accuracy of the system was not satisfactory, our results suggest that it might be useful in extracting and standardizing patients’ expressions related to symptoms from massive amounts of free text data instead of performing those procedures manually. After improving the extraction accuracy, we expect to utilize this system to detect the signals of ADRs from patients’ complaints in the future.

## Appendix

Multimedia Appendix 1 Search rules for the program.

## Abbreviations

ADE: adverse drug event
ADR: adverse drug reaction
AMED: Japan Agency for Medical Research and Development
CRF: conditional random fields
EHR: electronic health record
EMHD: electronic medication history data
FAERS: US Food and Drug Administration Adverse Event Reporting System
ICD-10: International Classification of Diseases, Tenth Revision
MedDRA: Medical Dictionary for Regulatory Activities
NLP: natural language processing
